# Effectiveness of epidural steroid injections in the treatment of lumbar radiculopathy: a systematic review and meta-analysis

**DOI:** 10.3389/fpain.2026.1904875

**Published:** 2026-08-21

**Authors:** Sebastian E. Moran, Samuel B. Kankam, Andrew O. Schindler, Sophia Dinh, Ryan Dinh, Neha Kulkarni, Sarah Voon, Audrey Demand, Paul J. Holman, Meng Huang, Sean Barber

**Affiliations:** 1 Department of Neurosurgery, Houston Methodist Hospital, Houston, TX, United States; 2 School of Engineering Medicine, Texas A&M University, Houston, TX, United States; 3 Biology Department, University of Houston, Houston, TX, United States; 4 Neuroscience Department, University of Texas, Austin, TX, United States

**Keywords:** caudal, epidural steroid injection, interlaminar, lumbar radicular pain, transforaminal

## Abstract

**Introduction:**

Epidural steroid injection (ESI) techniques have demonstrated effectiveness in improving short- and intermediate-term pain and disability outcomes; however, their efficacy may vary depending on the specific approach used. This meta-analysis aims to compare disability and pain outcomes among different ESI techniques - transforaminal (TFESI), interlaminar (ILESI), caudal (CESI), and combined approaches - in patients with lumbar radiculopathy.

**Methods:**

The authors searched PubMed/MEDLINE, Scopus, and Cochrane from inception to December 16, 2025, in accordance with PRISMA guidelines. Studies comparing disability and pain outcomes between different ESIs were assessed. Random-effects meta-analyses were performed to estimate the pooled mean difference between treatment groups at different follow-up timepoints. Sensitivity analyses and publication bias assessments were conducted.

**Results:**

In total, 31 studies comprising 2,452 patients were included. Compared with ILESI, TFESI demonstrated significantly greater pain reduction at 2 weeks and 1 month of follow-up, as well as superior improvements in both disability and pain at 3 and 6 months. Compared with CESI, TFESI showed superior disability improvements at 2 weeks and 1 month, with pain outcomes remaining superior at 1 and 3 months; however, both disability and pain outcomes plateaued by 6 months. At 12-month follow-up, TFESI demonstrated improved disability scores compared with CESI, with no significant difference in pain outcomes. Across all follow-up timepoints, the combined TFESI + CESI approach was associated with significantly greater improvements in both disability and pain relief compared with TFESI alone.

**Conclusions:**

TFESI demonstrated superior short- and intermediate-term improvements in disability and pain compared with ILESI, but its advantages over CESI remained inconsistent. Limited evidence suggests that the combined TFESI + CESI approach may offer additional benefits over TFESI alone. Further prospective randomized trials are warranted to better define the efficacy and long-term outcomes of the combined approach.

**Systematic Review Registration:**

https://www.crd.york.ac.uk/PROSPERO/view/CRD420261320804

## 1 Introduction

Epidural steroid injections (ESIs) have long been used as a first-line treatment for lumbar radiculopathy, with the goal of reducing inflammation at the affected nerve root and improving pain and function outcomes for affected patients (1, 2). Despite their widespread adoption, the clinical effectiveness of ESIs remains controversial. Inconsistencies in ESI efficacy result from the substantial heterogeneity in patient selection, underlying pathology, injection technique, outcome measures, and timing of outcome assessment that exists across studies (2–5). For example, lumbar radiculopathy, particularly when caused by disc herniation, sometimes improves spontaneously over time (2) and studies focusing just on short-term impacts may exaggerate the efficacy of treatment, whereas those examining longer follow-up periods may present diminished outcomes (1, 6). Most studies show that the benefits of ESIs are greatest in the short term, with effects fading after 6 to 12 months (2, 6, 7).

ESIs are commonly performed via interlaminar (ILESI), transforaminal (TFESI), or caudal (CESI) routes, each differing in target specificity, medication dispersion, and injectate volume requirements (1, 2, 8). Interlaminar injections deliver steroids into the posterior epidural space, relying on diffusion toward the ventral epidural compartment and dorsal root ganglion (2) and have varied effectiveness influenced by anatomic factors such as spinal stenosis or epidural scarring. In contrast, TFESIs deliver medication more selectively to the affected nerve root and ventral epidural space and are frequently used in cases of disc herniation or foraminal and lateral recess stenosis (1). Meanwhile, CESIs, while technically straightforward and sometimes perceived as safer, are less anatomically selective and typically require higher injectate volumes, potentially resulting in lower steroid concentration at the target level and reduced efficacy, particularly in chronic radiculopathy (1, 2).

Besides the approach trajectory, variation in steroid formulation, dose, and injectate volume between the ESI approaches has been reported (1, 3, 9); however, the dose–response relationships are not well established, with some studies suggesting that lower doses may provide comparable short-term relief to higher doses (2, 7). Similarly, particulate and nonparticulate steroids have not shown consistent differences in clinical effectiveness, although evidence supports nonparticulate steroids as a first-line treatment choice due to their enhanced safety profile (10). However, repeated injections over time may modulate ESI's effect contributing to the heterogeneity and uncertainties on treatment outcomes (11, 12).

Although numerous prior systematic reviews and meta-analyses have examined the conflicting evidence surrounding ESI efficacy (1, 4, 6, 7, 13–17), only a few have directly compared the various ESI approaches (1, 7, 14, 18–21). Moreover, the emerging literature on combined TFESI plus CESI vs. other approaches (22–24) necessitates an updated synthesis of the available data. Such an analysis is essential to further clarify the relative effectiveness of this combined strategy and to better inform tailored treatment selection for specific patient populations. This meta-analysis aims to examine the temporal variations in clinical outcomes following ESIs for lumbar radiculopathy and to compare these variations among interlaminar, transforaminal, caudal, and combined transforaminal plus caudal techniques.

## 2 Methods

This systematic review was conducted in accordance with the Preferred Reporting Items for Systematic Reviews and Meta-Analyses (PRISMA) 2020 (25), registered with PROSPERO under the number CRD420261320804 and available at https://www.crd.york.ac.uk/PROSPERO/view/CRD420261320804.

### 2.1 Study strategy

The authors searched PubMed/Medline, Scopus, and Cochrane for relevant studies from inception to 12/16/2025, without any language restrictions. The search strategy included the keywords “lumbar radiculopathy,” “sciatica,” “epidural steroid injection,” “transforaminal epidural steroid injection,” “interlaminar epidural steroid injection,” and “caudal epidural steroid injection.” Reference lists of relevant articles were also screened to identify additional studies. The full search syntax is shown in Supplementary Table S1.

### 2.2 Study selection process

The data from each electronic database were imported into Rayyan.ai. After removing duplicate publications, two reviewers independently screened the titles and abstracts to identify relevant studies. Full-text screening was then performed to determine which papers met the eligibility criteria. Any discrepancies were resolved by a third reviewer.

### 2.3 Eligibility criteria

We included studies that focused on patients with lumbar radiculopathy who received ESIs via the transforaminal, interlaminar, caudal, or combined transforaminal and caudal approaches. Studies were eligible for inclusion if they: (1) involved at least two comparison groups; (2) reported clinically relevant data on the effectiveness of the intervention for radicular pain across at least two ESI approaches; (3) described patients only with low back pain if radiculopathy was not specifically excluded, and (4) provided sufficient information regarding procedural technique, study methodology, and data analysis. Studies were excluded if they: (1) excluded patients with radiculopathy and only included neurogenic claudication; (2) did not specify ESI route used; (3) compared two variations of the same approach instead of two distinct routes; (4) did not report follow-up data on Oswestry disability index (ODI), visual analogue scale (VAS), numeric rating scale (NRS), numeric pain rating scale (NPRS) or visual numerical scale (VNS) scores; (5) lacked accessible full-text manuscripts; (6) were published in a language other than English; or (7) were non-original research articles, including case reports, case series with fewer than five patients, book chapters, review articles, letters to the editor, or conference abstracts.

### 2.4 Data extraction

Data extracted from each eligible study included the study population, study design, author name, year of publication, country, type of study, patient groups, mean age, sex distribution, intervention details (drug and dose), and periprocedural outcome measures. Outcome assessments included ODI and pain scores reported using various tools (VAS, NRS, NPRS, VNS) at different timepoints such as baseline, 2 weeks, 1 month, 3 months, 6 months and 12 months, as well as follow-up duration and reported complications.

### 2.5 Study assessments

For the quality assessment of the included studies, two distinct tools were applied depending on the study design. For non-randomized clinical trials, we used the Risk of Bias (RoB) tool for non-randomized studies (ROBIN-I) (26). This tool evaluates bias across seven domains: confounding, selection of participants, classification of interventions, deviations from intended interventions, missing data, measurement of outcomes, and selection of reported results. For randomized clinical trials (RCTs), RoB II tool was used to assess methodological validity and potential sources of bias (27). RoB II evaluates five key domains: the randomization process, deviations from intended interventions, missing outcome data, measurement of outcomes, and selection of the reported results. Two authors independently assessed each study and recorded their judgments. Any discrepancies between reviewers were resolved through discussion with a third, independent researcher.

### 2.6 Data synthesis

We followed the Cochrane Handbook for Systematic Reviews of Interventions to determine the appropriate effect sizes (28). For pain scores reported using different assessment tools (VAS, NRS, NPRS, and VNS), all values were converted to a standardized 0–10 scale, and standardized mean differences (SMDs; Hedges’ g) were calculated. For ODI and pain outcomes reported using the same assessment tool and scale, mean differences (MDs) from baseline to the various follow-up timepoints between comparison groups were calculated. All pooled estimates were derived using a random-effects model with Restricted Maximum Likelihood (REML) estimation. Heterogeneity was considered high when the Q-test *P*-value was <0.05 and the I^2^ statistic exceeded 40%. All pooled estimates were reported with corresponding 95% confidence intervals (CIs). Sensitivity analyses were performed using the leave-one-out (LOO) method to evaluate the robustness of the findings; however, LOO analyses were not conducted for comparisons with fewer than three studies at a given timepoint. Publication bias was assessed using funnel plots and Egger's test. All statistical analyses with *p* value <0.05 were considered statistically significant. Statistical analyses were performed with Stata version.17.0.

## 3 Results

### 3.1 Study selection process

**Figure 1:**
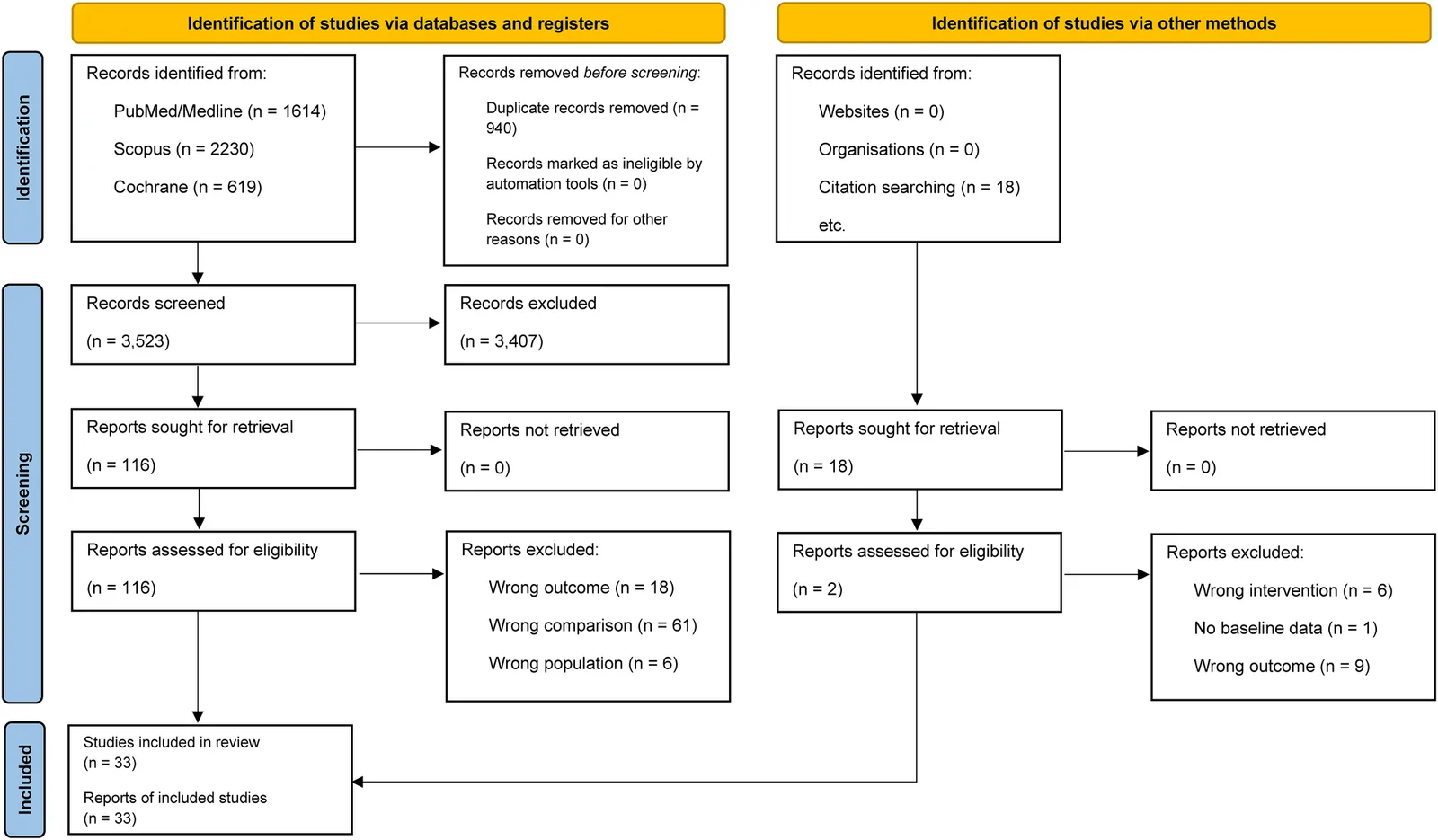
PRISMA 2020 flowchart illustrating the study selection process for the systematic review of ESI approaches.

The initial searches on December 16, 2025, identified 4,463 articles. After removing 940 duplicate records, 3,523 unique articles were left for screening. Titles and abstracts were initially screened and studies excluded based on the pre-determined exclusion criteria. Full texts of 116 potentially eligible studies were retrieved and screened. Of these, 86 were excluded and 30 met inclusion criteria. Also, 18 additional studies were identified by reference review, of which 3 met inclusion criteria. Thus, 33 studies satisfied the inclusion criteria and were incorporated into the systematic review and meta-analysis (22–24, 29–58). Figure 1 represents the study selection process.

### 3.2 Risk of bias

**Figure 2:**
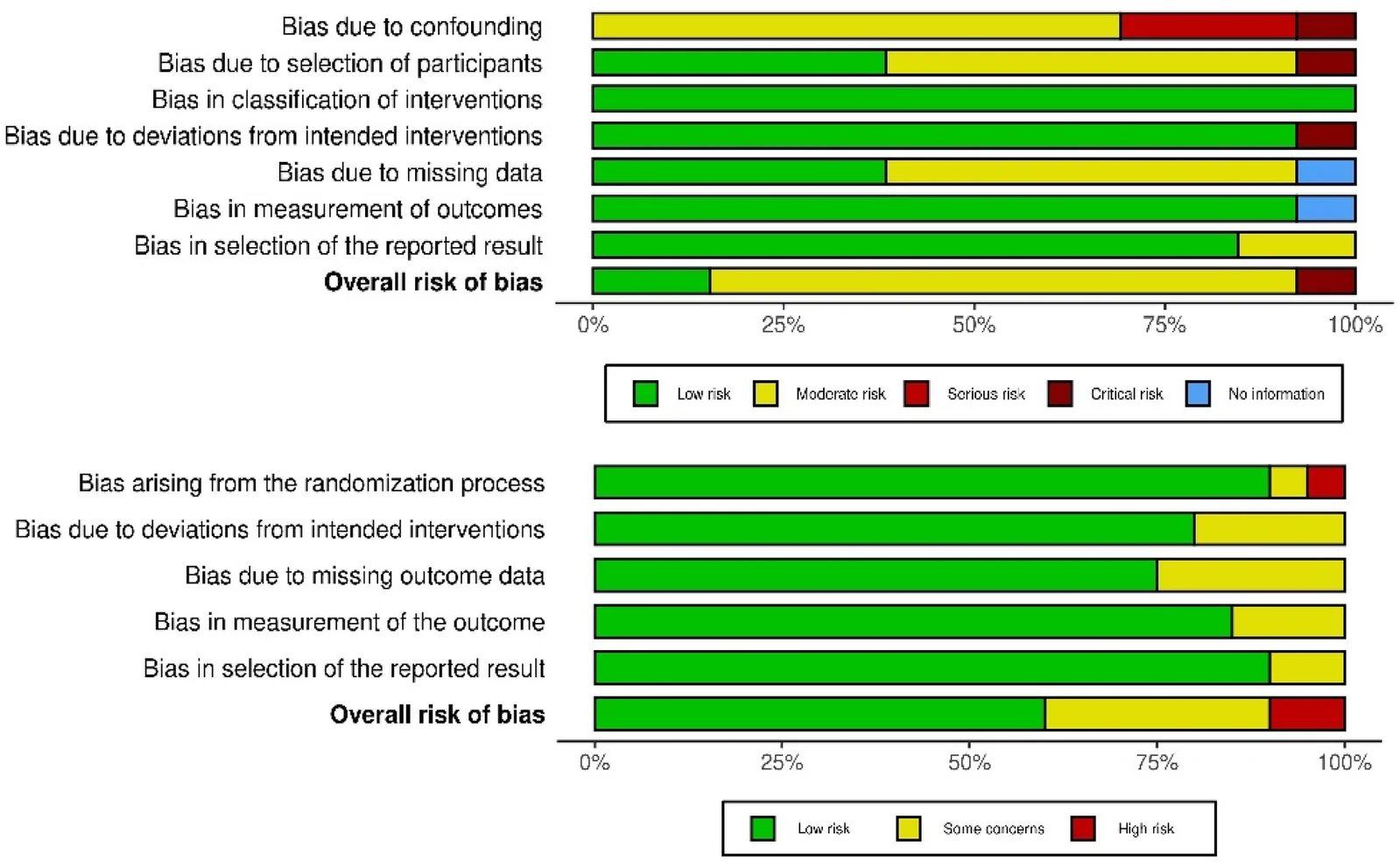
Risk of bias for non-randomized and randomized studies.

Risk-of-bias assessments of the 33 studies are shown on Figure 2. Fourteen studies were assessed as having a low risk (29, 33–37, 41, 44–46, 50, 51, 54, 56), sixteen a moderate risk (22–24, 31, 32, 38–40, 42, 48, 49, 52, 53, 55, 57, 58) and three a high risk of bias (30, 43, 47). Publication bias are presented in SR1.

### 3.3 Study characteristics

Of the 33 included studies, 19 were RCTs (29, 30, 32–38, 42–46, 48, 51, 54–56), 8 were retrospective cohort studies (22–24, 31, 41, 47, 52, 58), and 7 were prospective cohort studies (39, 40, 49, 50, 53, 57). The total sample size was 2,452 patients (range, 31–338 participants). Sex distribution was reported in 27 of 33 studies, with a cumulative total of 1,020 males and 1,014 females across reported cohorts. The range of reported group mean ages across studies was 33.83 to 64.6 years. The intervention groups included 17 studies which compared TFESI vs ILESI (29–44, 58), 10 studies TFESI vs CESI (45–54), and 3 studies TFESI vs TFESI + CESI (22–24). Also, 3 multi-arm studies included TFESI, ILESI, and CESI combined approaches (55–57). ESI injections were predominantly performed under fluoroscopic guidance, although 2 studies used CT guidance (39, 40). Thirteen studies also reported repeated injections (30–32, 36, 38, 39, 41, 43, 44, 47, 55–57).

In the TFESI vs ILESI group, 12 studies used particulate steroids, including triamcinolone (29, 33, 40, 43, 58), methylprednisolone (30–32, 34, 41), betamethasone (35, 37), while 4 studies used non-particulate dexamethasone (36, 38, 39, 42). Regarding triamcinolone dosing, 4 studies used 40 mg (29, 33, 43, 58), and 2 studies 80 mg (40, 44). For methylprednisolone, all studies used 80 mg except for one study (32). One study used 2 mL betamethasone (35), and another used 3 mL (37). Similarly, 3 studies used 5 mg of dexamethasone (36, 38, 42), while one study used 12 mg (Table 1) (39).

**Table 1:** Baseline demographics, epidural steroid injection characteristics, outcome assessment methods, and comparative outcomes between TFESI and ILESI groups.

Authors/ Year	Country	Study Design	Total No./Pt groups	Sex (M/F)/Age	Intervention drug/dose	Repeated Injection	Injection performed under imaging?	Outcome Evaluation Tool	Finding Summary
Gharu E, et al/2024 (29)	India	RCT	60/ G1: 30 G2: 30	G1: (NR)/NR G2: (NR)/ N4	G1: TFESI w/ 2 mL (40 mg/mL) of TC G2: ILESI w/ 2 mL (40 mg/mL) of TC^§^	NR	Fluoro	ODI, VAS, mJOA	F/U duration: 6 months It was found that there was no significant difference seen in ODI, and VAS score at 1 month, 3 months, and 6 months between TFESI and ILESI. Complications: NR
Kawu A./2012 (30)	Nigeria	RCT	47/ G1: 25 G2:22	G1:(18M, 7F)/46.9 ± 10.7 G2:(16M, 6F:)/48.3 ± 11.3	G1: TFESI w/ 80 mg MP G2: ILESI w/ 80 mg MP	G1: 9 G2: 11	Mobile x-ray	ODI, VAS	F/U duration: 6 months Repeated in Group 1 had statistically significant improvements in ODI and VAS scores compared to Group 2. Complications: NR
Schaufele M, et al/2006 (31)	USA	Retro	40/ G1: 20 G2: 20	G1: (NR)/ NR G2: (NR)/ NR	G1: TFESI w/ 80 mg MP G2: ILESI w/ 80 mg MP	G1: 9 G2: 8	Fluoro	VNS	F/U duration: 2–3 weeks Fourteen patients (70%) had an improvement of 2 points or more on the VNRS scale in the TFESI group, compared to 9 (45%) in the ILESI group. Complications: NR
Rados I, et al/2011 (32)	Croatia	RCT	64/ G1:32 G2:32	G1:(20M, 12F)/48.8 G2:(21M, 11F)/ 49.2	G1: TFESI w/ 40 mg MP G2: ILESI w/ 80 mg MP	All patients three ESI	Fluoro	VAS, ODI, GPE questionnaire	F/U duration: 6 months No statistically significant difference between two groups, although half-dose of steroids delivered via TF route provided somewhat better long-term pain relief and functional capacity improvements. Complications: NR
Kumar D, et al/2023 (33)	India	RCT	60/ G1: 30 G2: 30	G1:(23M,7F:)/34.8 ± 8.2 G2:(24M,6F:)/33.8 ± 8.5	G1: TFESI w/ 1 mL (40 mg/mL) of TC G2: ILESI w/ 1 mL (40 mg/mL) of TC	NR	Fluoro	NRS, VAS	F/U duration: 1 month. Patients who received triamcinolone acetate via TF route experienced greater pain reduction in terms of NRS and VAS at the second week. Complications: NR
Makkar J, et al/2019 (34)	India	RCT	65 (61 analyzed)/ G1: 20 G2: 21 G3: 20	G1: (11M,12F:)/37.7 ± 6.7 G2: (12M, 9F)/42.7 ± 7.5 G3: (7M,14F)/ 41.2 ± 7.4	G1: TFESI w/ 80 mg MP G2: ILESI (Midline) w/ 80 mg MP G3: ILESI (Parasagittal) w/ 80 mg MP	NR	Fluoro	ODI, VAS, serum osteocalcin levels, DEXA scan	F/U duration^#^: 6 months. Group 1 and 3 had significantly lower ODI and VAS scores compared to Group 2 but were not significantly different from each other. Complications: NR
Soliman A, Fahmy M./2018 (35)	Egypt	RCT	40/ G1: 20 G2: 20	G1: (6M,14F:)/ 37.5 ± 10.8 G2: (11M,9F)/35 ± 9.9	G1: TFESI w/ 2 mL BMZ G2: ILESI w/ 2 mL BMZ	NR	Fluoro	ODI, VAS	F/U duration: 48 weeks The only statistically significant improvement was in VAS for leg pain and the ODI in Group 1. Complications: G1: 3 cases of increased pain at injection site. G2: 1 case of vasovagal reaction, 1 case of dural puncture and post puncture headache.
Hong J, et al/2017 (36)	South Korea	RCT	72/ G1: 31 G2: 41	G1: (15M,16F)/59.9 ± 13.1 G2: (15M, 26F)/60.2 ± 12	G1: TFESI w/ 5 mg DEX G2: ILESI (parasagittal) w/ 5 mg DEX	Injections were done twice in both groups.	Fluoro	ODI, NRS	F/U duration: 2 weeks. Both the PIL and TF approach produced similar clinically significant improvements in pain and level of disability. Complications: None
De Barcellos Z, et al/2015 (37)	Brazil	RCT	40/ G1:20 G2:20	G1: (7M, 13F:)/ 48.85 G2: (10M, 10F:)/ 50.5	G1: TFESI w/ 3 mL (40 mg/mL) BMZ G2: ILESI w/ 3 mL (40 mg/mL) BMZ	NR	Fluoro	VAS	F/U duration: 3 months The techniques differ only in the post-blocking period of 21 days and overall post-blocking, with significance of *p* = 0.022 and *p* = 0.027, respectively. Complications: G1: 1 case of lower back pain. G2: 1 case of headache.
Choi E, et al./2021 (38)	South Korea	RCT	59/ G1: 29 G2:30	G1:(12M,17F:)/64.6 ± 12.5 G2:(17M,13F:)/59.1 ± 13.7	G1: TFESI w/ 5 mg DEX G2: ILESI (oblique approach) w/ 5 mg DEX	Injections were done up to 4 times in each group.	Fluoro	ODI, RMDQ, VAS	F/U duration: 3 months There were no significant differences between the groups in the VAS, and ODI scores during the 12-week period. Complications: None
Bise S, et al/2023 (39)	France	Pros	237/ G1: 71 G2:166	G1:(33M, 38F)/56 ± 13 G2:(85M, 81F)/ 51 ± 16	G1: TFESI w/ 12 mg DEX G2: ILESI w/ 12 mg DEX	G1: 1 G2: 8	CT	ODI, VAS	F/U duration: 5 years No significant differences were observed between the two approaches in VAS or ODI decreases.^α^ Complications: None
Rahatli F, et al/2017 (40)	Turkey	Pros	87/ G1:23 G2:64	G1: (NR)/73 G2: (NR)/68	G1: TFESI w/ 80 mg TC G2: ILESI w/ 80 mg TC	NR	CT	VAS	F/U duration: 6 months Transforaminal group showed slightly better pain relief in all terms, but the difference was not statistically significant. Complications: 60 cases of pain at injection site/motor weakness and numbness but study didn't specify in which groups.
Smith C, et al/2010 (41)	USA	Retro	38/ G1:19 G2:19	G1: (NS)/67.7 G2: (NS)/67.1	G1: TFESI w/ 80 mg MP G2: ILESI w/ 80 mg MP	G1: 16 (2.58) G2: 14 (2.47)	Fluoro	VAS	F/U duration: 3 years. There was no statistically significant difference between the two groups.^β^ Complications: NR
Thomas E, et al/2003 (42)	France	RCT	31/ G1: 15 G2:16	G1:(8M,7F)/49.8 ± 13.9 G2:(5M,11F)/51.3 ± 17	G1: TFESI w/ 5 mg DEX G2: ILESI w/ 5 mg DEX	NR	Fluoro	VAS, Schober's index, Finger-to-floor distance, straight leg raise test, DPQ, RMLBPQ	F/U duration: 6 months Pain relief with TFESI was significantly better at 30 days and 6 months when compared to ILESI. Complications: NR
Yilmaz A, Küçükbingöz C./ 2025 (58)	Turkey	Retro	124/ G1: 64 G2: 60	G1: (0M,64F)/47.8 ± 13.8 G2: (52M,8F)/50.8 ± 15.7	G1: TFESI w/ 40 mg TC G2: ILESI w/ 40 mg TC	NR	Fluoro	ODI, VAS, DN4, patient satisfaction scale	F/U duration: 6 months TFESI was superior to ILESI with statistically significant differences in VAS and ODI at 6 months. Complications: Procedure-related complication rates were 14.10% (9/64) in TFESI and 13.33% (8/60) in ILESI. The most common events were transient paresthesia or needle trauma.
Gharibo C, et al/ 2011 (43)	USA	RCT	42 (38 included in final analysis)/ G1: 20 G2: 18	G1: (11M,9F:)/NR G2: (13M, 5F)/ NR	G1: TFESI w/ 40 mg TC G2: ILESI w/ 40 mg TC	G1: 1 G2: 3	Fluoro	ODI, NRS, Depression assessment and physical therapy tolerance	F/U duration: 10–16 days. Group 1 had a greater reduction in NRS. Complications: NR
Kaur S, et al/2017 (44)	India	RCT	60/ G1:20 G2:20	G1:(10M,10F)/50.6 ± 15.0 G 2:(14M,6F:)/45.1 ± 13.1	G1: TFESI w/ 80 mg TC w/ 1,500 U hyaluronidase and 50 mg tramadol G2: Midline ILESI w/ 80 mg TC w/ 1,500 U hyaluronidase and 50 mg tramadol	G1: 6 G2: 10	Fluoro	VAS, Quebec disability score, depression score	F/U duration: 3 months. Repeated injection -yes. On intergroup comparison, the difference in improvement of VAS score noted was statistically insignificant. Complications: NR

RMDQ; Roland Morris disability questionnaire, ODI; Oswestry disability index, VAS; visual analogue scale, NRS; numeric rating scale, NR; not reported, MS; months; GPE; global perceived effect questionnaire, DPQ; Dallas pain questionnaire, RMLBPQ; Roland-Morris low back pain questionnaire, DN4; neuropathic pain component, mJOA; modified Japanese Orthopedic Association scale, F/U; follow-up, Fluoro: fluoroscopic guidance, CT; computed tomography guidance, BMZ; betamethasone; MP; methylprednisolone, TC; triamcinolone, DEX; dexamethasone.

§ Both groups also had 7 mL of normal saline added to the injections used.

β The follow up for patient reported outcomes was 4–6 weeks but all patients had a 3-year follow-up minimum that was mainly used in the article to report the need for repeat injections.

α Only 96 patients had 5 years follow-up. In the spreadsheet we have values up to 6 months.

# 4 patients lost to follow-up and not analyzed.

In the TFESI vs CESI group, the majority of the studies used particulate steroids, including triamcinolone (46, 48–50) and methylprednisolone (51, 52). The triamcinolone dose varied between 20 mg (48) and 40 mg (46, 49, 50). Likewise, methylprednisolone dose varied from 40 mg (51) to 80 mg (52–54). Two studies used non-particulate dexamethasone one at 10 mg (47) and the other at 12 mg (45) (Table 2).

**Table 2:** Baseline demographics, epidural steroid injection characteristics, outcome assessment methods, and comparative outcomes between TFESI and CESI groups.

Authors/ Year	Country	Study Design	Total No./Pt groups	Sex (M/F)/Age	Intervention drug/dose	Repeat Injection	Injection performed under imaging?	Outcome Evaluation Tool	Finding Summary
Ozturk E, et al/2023 (45)	Turkey	RCT	60/ G1: 30 G2: 30	G1:(15M,15F)/ 38.86 G2: (18M,12F)/ 41.86	G1: TFESI w/ 12 mg DEX G2: CESI w/ 12 mg DEX*	NR	Fluoro	ODI, NRS	F/U duration: 3 months Treatment success rate at 3rd month was 77% for the CESI group and 73% for the TFESI group without any significant difference between the groups. Complications: NR
Celenlioglu A, et al/2022 (46)	Turkey	RCT	56/ G1: 30 G2:26	G1:(21M,9F)/ 51.8 ± 12.6 G2: (17M, 9F)/ 53.4 ± 11.2	G1: TFESI w/ 40 mg of TC, 2 mL of saline G2: CESI w/ 40 mg of TC, 7 mL of saline	None	Fluoro	ODI, NRS	F/U duration: 3 months Although both methods have similar treatment success rates, TFESI seems to be a more effective treatment method in reducing disability at 3-week follow-up. Complications: G1: 4 cases of Intravascular contrast filling pattern, 2 cases of vasovagal reaction, 5 cases of temporary motor block. G2: 1 case of intravascular contrast filling pattern, 2 cases of vasovagal reaction, 2 cases of temporary motor block
Song J, et al/2021 (47)	South Korea	Retro	49/ G1:28 G2: 21	G1: (9M,19F)/ 59.1 ± 10.2 G2: (6M,15F)/ 60.5 ± 10.4	G1: TFESI w/10 mg DEX G2: CESI w/ 10 mg DEX	G1: 9 G2: 7	Fluoro	ODI, VNS	F/U duration: 6 months No meaningful difference was present between groups in terms of treatment success rate at every time point. Complications: G1: 4 cases of intravascular contrast spread, 1 case of vasovagal reaction, 3 cases of transient headache, 9 cases of transient pain exacerbation. G2: 4 cases of vasovagal reaction, 4 cases of transient headache, and 3 cases of transient pain exacerbation.
Imani F, et al/2024 (48)	Iran	RCT	50/ G1: 25 G2: 25	G1: (11M,14F)/51.7 ± 16.7 G2:(12M,13F)/53.9 ± 11.0	G1: TFESI w/ 20 mg TC G2: CESI w/ targeted catheter w/ 20 mg TC	None	Fluoro	ODI, VAS	F/U duration: 3 months No significant difference between the groups. Complications: None
Muthu S, et al/2025 (49)	India	Pros	54/ G1: 28 G2: 26	G1:(10M,18F:)/ 38.9 ± 3.9 G2:(12M, 14F)/ 36.1 ± 4.1	G1: TFESI w/ 2 mL (40 mg/mL) of TC G2: CESI w/ 2 mL (40 mg/mL) of TC	NR	Fluoro	ODI, NPRS	F/U duration: 6 months The pain and functional score reduction between the two groups was comparable at every follow-up. Increased fluoroscopy use in TF group than Caudal group Complications: None
Iqbal M, et al/2020 (50)	Pakistan	Pros	96/ G1: 48 G2: 48	G1:(29M,19F)/42 ± 8.4 G2:(35M,13F)/43.0 ± 9.3	G1: TFESI w/ 40 mg TC G2: CESI w/ 40 mg TC	NR	Fluoro	NRS	F/U duration: 3 months There was a reduction in mean pain score from baseline to 12 weeks in both the groups with statistical significance more in Group 1 compared to Group 2. Complications: NR
Karamouzian S, et al/2014 (51)	Iran	RCT	30/ G1: 15 G2:15	G1:(7M, 8F)/47.3 ± 9.5 G2:(7M, 8F)/48.3 ± 11.7	G1: TFESI w/40 mg MP G2: CESI w 40 mg MP	NR	Fluoro	NRS, standing tolerance, Walking tolerance	F/U duration: 6 months No statistically significant difference was found between the 2 groups for NPS, walking tolerance, nor standing tolerance. Complications: G2: 1 case of temporary paraparesis.
Noushad C, et al/2025 (52)	India	Retro	60/ G1: 30 G2: 30	G1:(17M,13F)/43.5 ± 8.3 G2:(14M, 16F)/40.2 ± 8.9	G1: TFESI w/80 mg MP G2: CESI w/80 mg MP	NR	Fluoro	ODI, VAS, CORI, MRM	F/U duration: 6 months By 6 months, the caudal group demonstrated significantly better outcomes across all parameters. Complications: NR
Gupta A, Bhat B./ 2020 (53)	India	Pros	40/ G1: 20 G2: 20	G1:(12M,8F)/53.8 G2:(14 M, 6F)/55.6	G1: TFESI w/80 mg MP w/ 4 mL bupivacaine and 4 mL saline G2: CESI w/ 80 mg MP w/ 4 mL bupivacaine and 4 mL saline	NR	x-ray projection	ODI, VAS	F/U duration: 12 months Mean VAS and ODI scores were lower with statistical significance in Group 1 except at the 1-year data point. Complications: NR
Garg S, et al. 2022 (54)	India	RCT	100/ G1: 50 G2: 50	G1: NR/44.5 G2: NR/ 44.5	G1: TFESI w/80 mg MP w/ 1 mL bupivacaine G2: CESI w/ 80 mg MP w/ 4 mL bupivacaine and 4 mL saline	NR	Fluoro	ODI, VAS	F/U duration: 12 months Mean VAS and mean ODI score for the patients of Group 1 was significantly lower in comparison to the patients of group 2 at post-last injection, 15 days post-last injection, 1-month post-last injection and 3 months post-last injection time interval. Complications: G1: 3 cases of headache. G2: 2 cases of injection site pain, sweating, and transient drowsiness

G1; group 1, G2; group 2, Pros; Prospective study, Retro; Retrospective study, ODI; Oswestry disability scale, VAS; visual analogue scale, VNS; verbal numeric scale, NPRS; numeric pain rating scale, MS; months, CORI; Clinical Outcome Rating Index, MRM; Modified Roland-Morris, MP; methylprednisolone, TC; triamcinolone, DEX; Dexamethasone, Fluoro: Fluoroscopic guidance, NR; not reported.

* Group 1 uses 1 mL of saline in the injection; Group 2 uses 2 mL of saline.

In the TFESI vs TFESI + CESI group, particulate methylprednisolone was used at doses of 20 mg (23) and 40 mg (24). (Table 3) Among studies comparing all three ESI approaches, 2 studies used particulate triamcinolone 40 mg (55, 56), while one study used non-particulate dexamethasone (Table 4) (57).

**Table 3:** Baseline demographics, epidural steroid injection characteristics, outcome assessment methods, and comparative outcomes between TFESI and TFESI + CESI groups.

Authors/ Year	Country	Study Design	Total No./ Pt groups	Sex (M/F)/Age	Intervention drug/dose	Repeat injection	Injection performed under imaging?	Outcome Evaluation Tool	Finding summary
Evran S, et al/2021 (22)	Turkey	Retro	71/ G1: 32 G2: 39	G1: (14M,18F)/49.7 ± 12.5 G2: (19M,20F)/53.6 ± 13.0	G1: TFESI G2: TFESI + CESI^‡^	NR	Fluoro	ODI, VAS	F/U duration: 6 months The mean of all VAS leg, VAS back, and ODI scores were found to be lower in the TFESI + CESI group than the only TFESI group at each third-week, third month, and sixth-month controls, and these differences were statistically significant. Complications: None
Kircelli A, et al/2018 (23)	Turkey	Retro	104/ G1: 52 G2: 52	G1: (13M, 39F)/ 62.4 ± 15.5 G2: (15M, 37F)/ 57.6 ± 15.7	G1: TFESI w/ 40 mg MP G2: TFESI w/ 20 mg MP + CESI w/ 20 mg MP and 25 mL of 0.9% of sodium chloride	NR	Fluoro	VNS, NASS, EQ-5D	F/U duration: 12 months Improvement in the VNS scores was significantly higher in the TFSI + CESI group. Complications: None
Baran O, Evran S./2020 (24)	Turkey	Retro	99/ G1: 48 G2:51	G1: (19M, 29F)/ 47.0 ± 11.2 G2: (20M, 31F)/ 45.3 ± 9.2	G1: TFESI w/ 40 mg MP G2: TFESI w/ 40 mg MP + CESI w/ 40 mg MP and 20 ccs of 0.9% sodium chloride	NR	Fluoro	ODI, VAS	F/U duration: 6 months Combined TFESI and CESI for multi-level lumbar disc disease provided significant improvement in pain management and functional capacity compared with TFESI alone. Complications: 4 cases of dizziness, not specified in which group.

ODI; Oswestry disability scale, VAS; visual analogue scale; G1; group 1, G2; group 2, TFESI; transforaminal epidural steroid injection, CESI; caudal epidural steroid injection, F/U; follow-up, VNS; verbal numeric scale, Pros; Prospective study, Retro; Retrospective study, MS; months, NASS; North American Spine Society pain satisfaction index, EQ-5D; EuroQol five dimensions quality of life scores, MP; methylprednisolone, Fluoro: Fluoroscopic guidance, NR; not reported.

‡ All the TFESIs and CESIs contained 40 mg of methylprednisolone. The CESIs also contained 17 ccs of 0.9% sodium chloride

**Table 4:** Baseline demographics, epidural steroid injection characteristics, outcome assessment methods, and comparative outcomes between TFESI, ILESI and CESI groups.

Authors/ Year	Country	Study Design	Total No./ Pt groups	Sex/Age	Intervention drug/dose	Repeat Injection	Injection performed under imaging?	Outcome Evaluation Tool	Finding summary
Kamble P, et al/2016 (55)	India	RCT	90/ G1: 30 G2: 30 G3: 30	G1: NR/ NR G2: NR/NR G3: NR/ NR	G1: TFESI w/40 mg TC G2: ILESI w/ 40 mg TC G3: CESI w/ 40 mg TC	G1: 4 G2: 3 G3: 5	Fluoro	ODI, VAS	F/U duration: 6 months The change in pain scores was statistically different at 1- and 6-month intervals such that a higher change was observed by transforaminal route as compared to the other two. Complications: NR
Ackerman W, Mahmood A./2007 (56)	USA	RCT	90/ G1: 30 G2: 30 G3: 30	G1:(20M,10F)/34 ± 5 G2:(21M,9F)/39.2 ± 6 G3:(19M,11F)/36.4 ± 4	G1: TFESI w/40 mg TC and 4 mL of saline G2: ILESI w/ 40 mg TC and 4 mL of saline G3: CESI w/ 40 mg TC and 19 mL of saline	Yes, but NR	Fluoro	ODI, NRS, OLBPS, BDS	F/U duration: 6 months Pain relief was significantly more effective with TF injections. Complications: None
Llevot J, et al/2023 (57)	Spain	Pros	99/ G1: 23 G2: 18 G3: 58	G1: NR/ NR G2: NR/NR G3: NR/ NR	G1: TFESI w/ 8 mg DEX G2: ILESI w/ 12 mg DEX G3: CESI w/ 12 mg DEX	Yes, but NR.	Radioscopic control	VAS, ICIDH-1	F/U duration: 3 months Transforaminal approach was superior to caudal or interlaminar. Complications: None

ODI; Oswestry disability index, OLBPS; Oswestry low back pain scale, BDS; Beck depression scores; ICIDH-1: WHO-modified International Classification of Impairments, Disabilities and Handicaps, VAS; visual analogue scale, NR; not reported, MS; months, TFESI; transforaminal epidural steroid injection, ILESI; intralaminar epidural steroid injection, CESI; caudal epidural steroid injection, Pros; Prospective study, Retro; Retrospective study, TC; triamcinolone, DEX; Dexamethasone, Fluoro: Fluoroscopic guidance.

Most studies assessed functional outcomes with ODI; however, peri-procedure pain outcomes were assessed via VAS (22, 24, 29, 30, 32–35, 37–42, 44, 48, 52–54, 58), NRS (36, 43, 45, 46, 50, 51), NPRS (49) and VNS (23, 31, 47). Twelve studies additionally evaluated other outcomes using various assessment tools, including mJOA (29), GPE questionnaire (32), serum osteocalcin levels (34), RMDQ (38), DPQ (42), RMLBPQ (42), DN4 (58), Quebec disability score (44), standing tolerance (51), walking tolerance (51), CORI (52), MRM (52), NASS (23), EQ-5D (23), OLBPS (56), BDS (44, 56), and ICIDH-1 (57). Follow up duration was varied, with outcomes assessed between 3 months and 6 months in several studies (22, 24, 29, 30, 32, 34, 37, 38, 40, 42, 44–52, 55–58), while others reported short-term follow-up of a few weeks (31, 33, 36, 43) and longer-term follow-up ranging from 1 to 5 years (23, 35, 39, 41, 53, 54). ESI associated complications were reported by 9 studies (24, 35, 37, 40, 46, 47, 51, 54, 58).

### 3.4 Main outcomes

#### 3.4.1 Baseline comparisons

**Figure 3:**
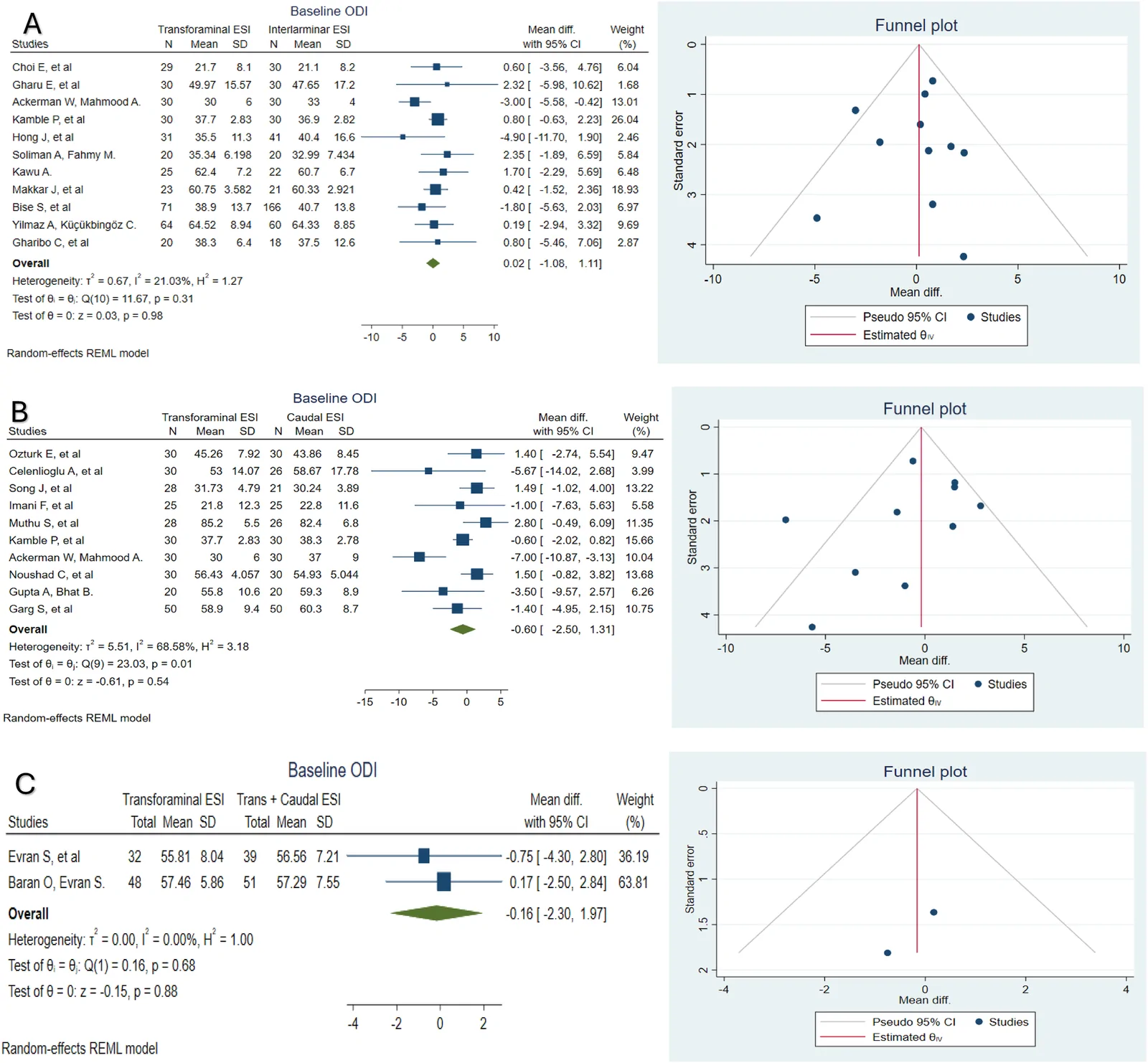
Baseline ODI scores among ESI groups.

**Figure 4:**
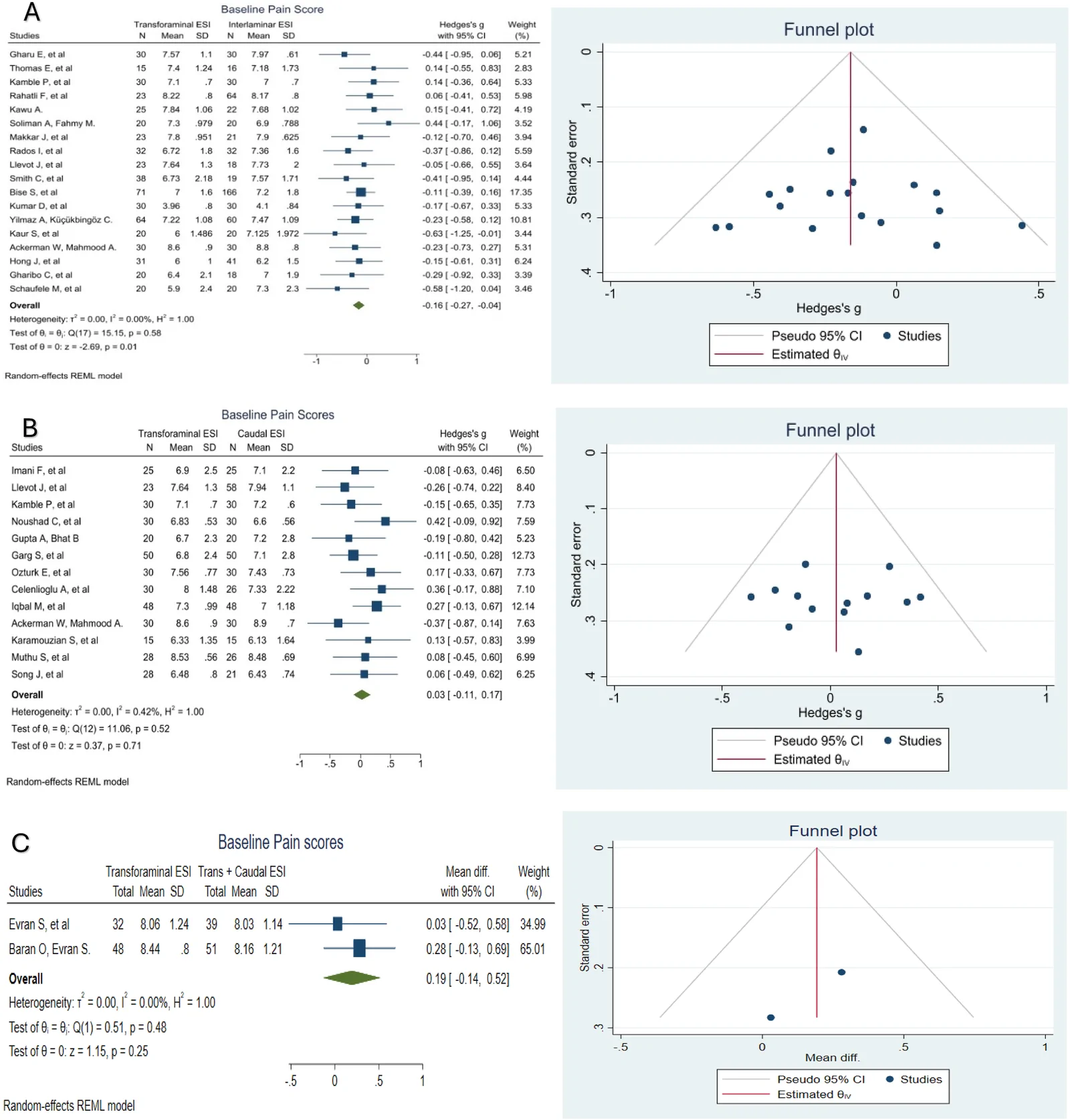
Baseline pain scores among ESI groups.

Across all analyses, baseline ODI and pain scores were comparable between injection approaches. Regarding ODI, TFESI did not differ from ILESI (MD: 0.02; 95% CI: −1.08 to 1.11; I^2^ = 21.03%) or CESI (MD: −0.60; 95% CI: −2.50 to 1.31; I^2^ = 68.58%), and it was also similar to TF + CESI (MD: −0.16; 95% CI: −2.30 to 1.97; I^2^ = 0%) (Figure 3). Although pain scores at baseline showed significant different between TFESI and ILESI (SMD: −0.16; 95% CI: −0.27 to −0.04; I^2^ = 0%), it was similar between TFESI and CESI (MD: 0.03; 95% CI: −0.11 to 0.17; I^2^ = 0.42%), or TFESI and TF + CESI (MD: 0.19; 95% CI: −0.14 to 0.52; I^2^ = 0%) (Figure 4).

#### 3.4.2 Early follow-up outcomes (2 weeks to 1 month)

**Figure 5:**
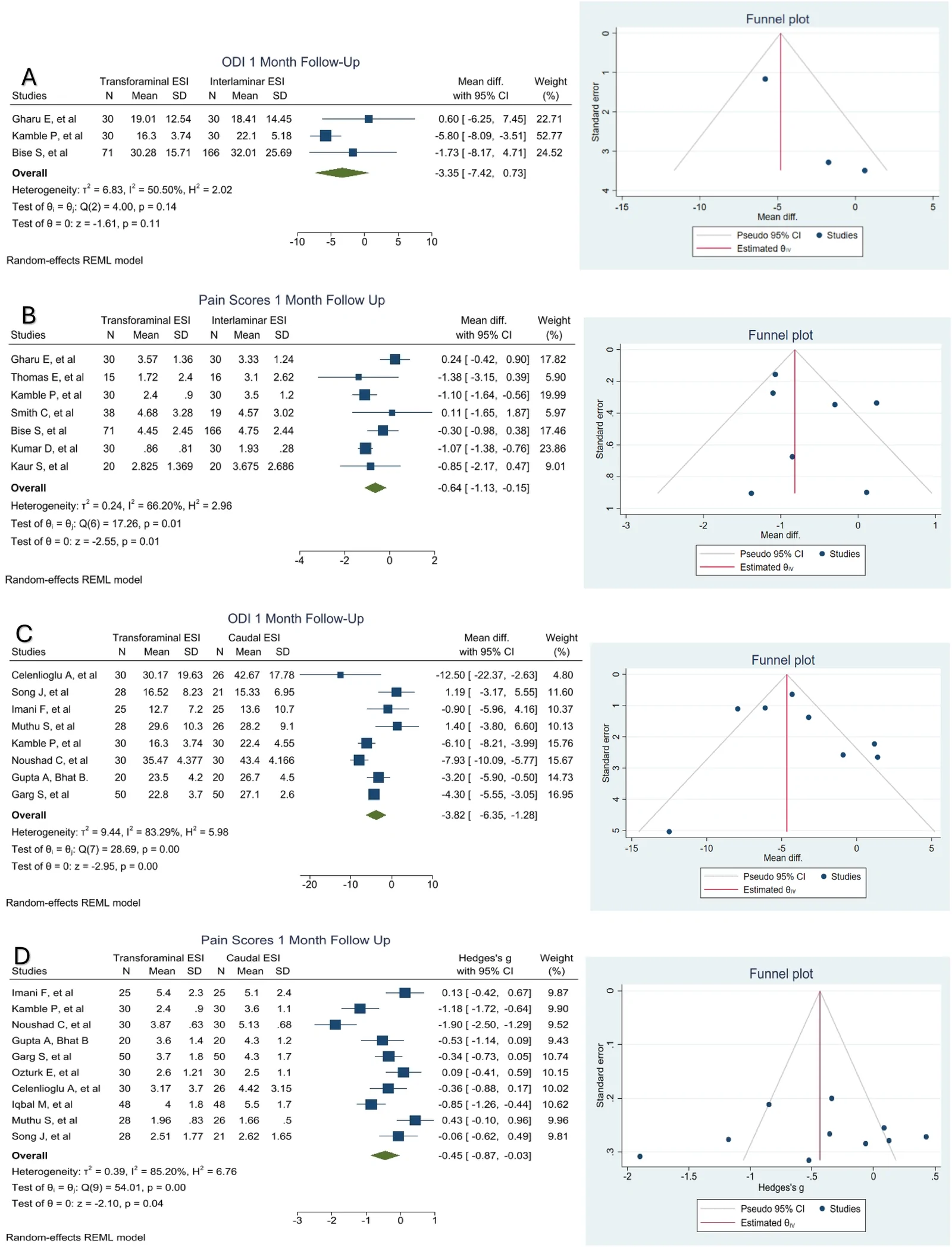
One month follow up; **(A)** ODI scores between TFESI vs ILESI; **(B)** Pain scores between TFESI vs ILESI; **(C)** ODI scores between TFESI vs CESI; **(D)** Pain scores between TFESI vs CESI.

At two weeks, TFESI and ILESI had no difference in ODI scores (MD: −0.33; 95% CI: −4.18 to 3.52; I^2^ = 23.24%) but significantly decreased in pain scores with TFESI compared to ILESI (MD: −0.73; 95% CI: −1.14 to −0.32; I^2^ = 70.75%) (Supplementary Figure S1). TFESI showed significant improvement in ODI scores (MD: −4.34; 95% CI: −7.64 to −1.05; I^2^ = 77.33%) compared to the CESI approach but no difference in pain outcomes (MD: −0.77; 95% CI: −1.78 to 0.24; I^2^ = 92.92%) at 2 weeks follow-up (Supplementary Figure S2). At one month follow-up, ODI scores were comparable between TFESI and ILESI approaches (MD: −3.35; 95% CI −7.42 to 0.73; I^2^ = 50.50%), although compared to ILESI, TFESI resulted in statistically significant improvement in pain scores (MD: −0.64; 95% CI −1.13 to −0.15; I^2^ = 66.20%). TFESI showed sustained improvement in ODI scores (MD: −3.82; 95% CI: −6.35 to −1.28; I^2^ = 83.29%) and pain scores (SMD: −0.45; 95% CI: −0.87 to −0.03; I^2^ = 85.20%) compared to CESI (Figure 5). Similarly, the combined TF + CESI approach significantly greater improvement in both ODI (MD: 5.98; 95% CI: 4.19 to 7.76; I^2^ = 0%) and pain scores (MD: 1.73; 95% CI: 0.56 to 2.89; I^2^ = 89.7%) compared with TFESI alone (Supplementary Figure S3).

#### 3.4.3 Intermediate follow-Up outcomes (3 months)

**Figure 6:**
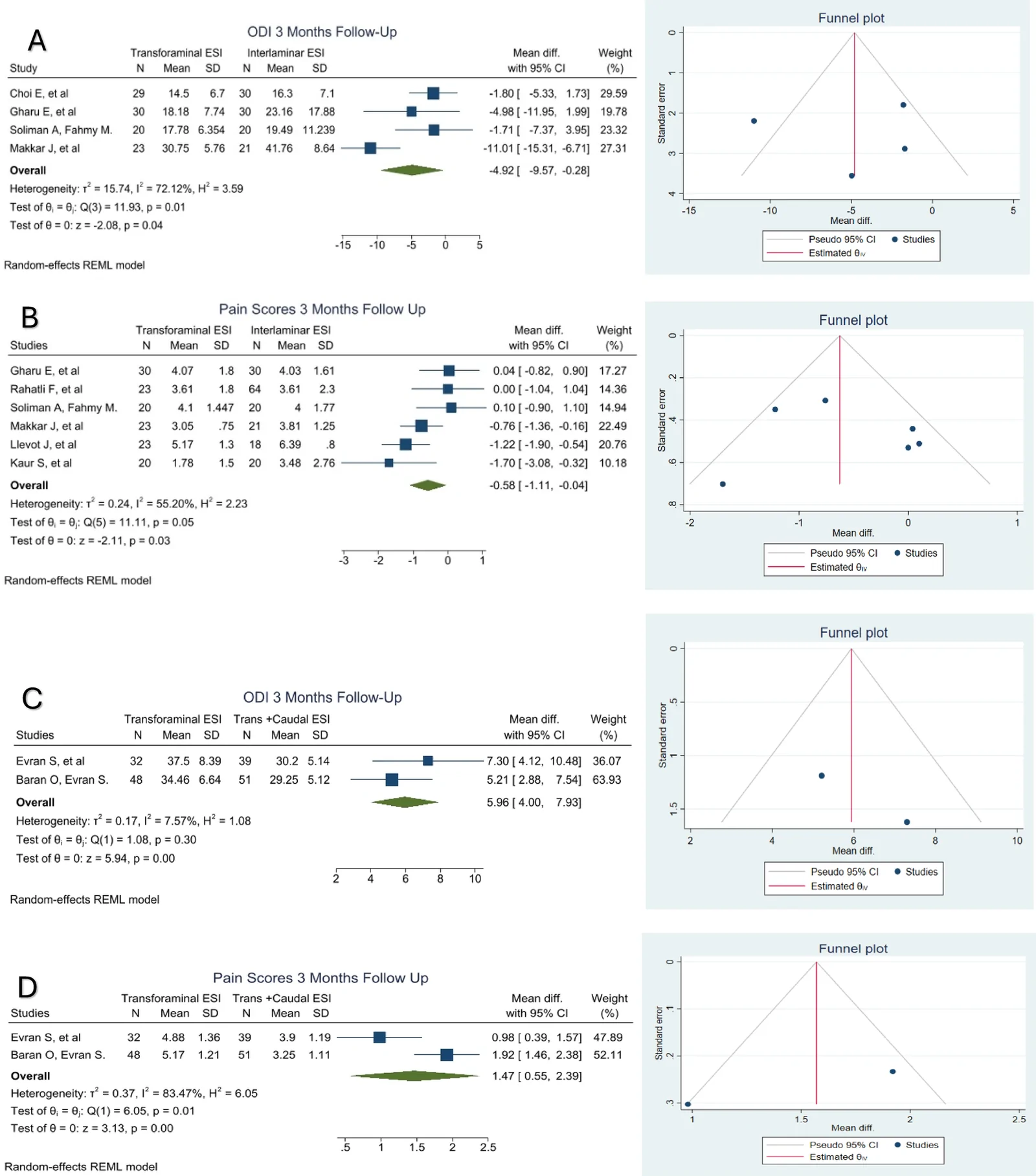
Outcomes at 3 months; (A) ODI scores between TFESI vs ILESI; (B) Pain scores between TFESI vs ILESI; (C) ODI scores between TFESI vs TFESI +CESI; (D) Pain scores between TFESI vs TFESI +CESI.

At 3-month follow-up, TFESI demonstrated improvement in ODI scores (MD: −4.92; 95% CI: −9.57 to −0. 28; I^2^ = 72.12%) and pain scores (MD: −0.58; 95% CI: −1.11 to −0.04; I^2^ = 55.20%) compared to the ILESI approach. Similarly, compared to CESI, TFESI showed reduced pain scores (SMD: −0.49; 95% CI: −0.96 to −0.02; I^2^ = 87.81%) but comparable ODI scores (MD: −0.20; 95% CI: −3.79 to 3.40; I^2^ = 86.45%) (Supplementary Figure S4). The combined TFESI + CESI technique was also associated with significantly greater improvements in ODI scores (MD: 5.96; 95% CI: 4.00 to 7.93; I^2^ = 7.57%) and pain scores (MD: 1.47; 95% CI: 0.55 to 2.39; I^2^ = 83.47%) relative to TFESI alone (Figure 6).

#### 3.4.4 Long-term follow-up outcomes (6 months-12 months)

**Figure 7:**
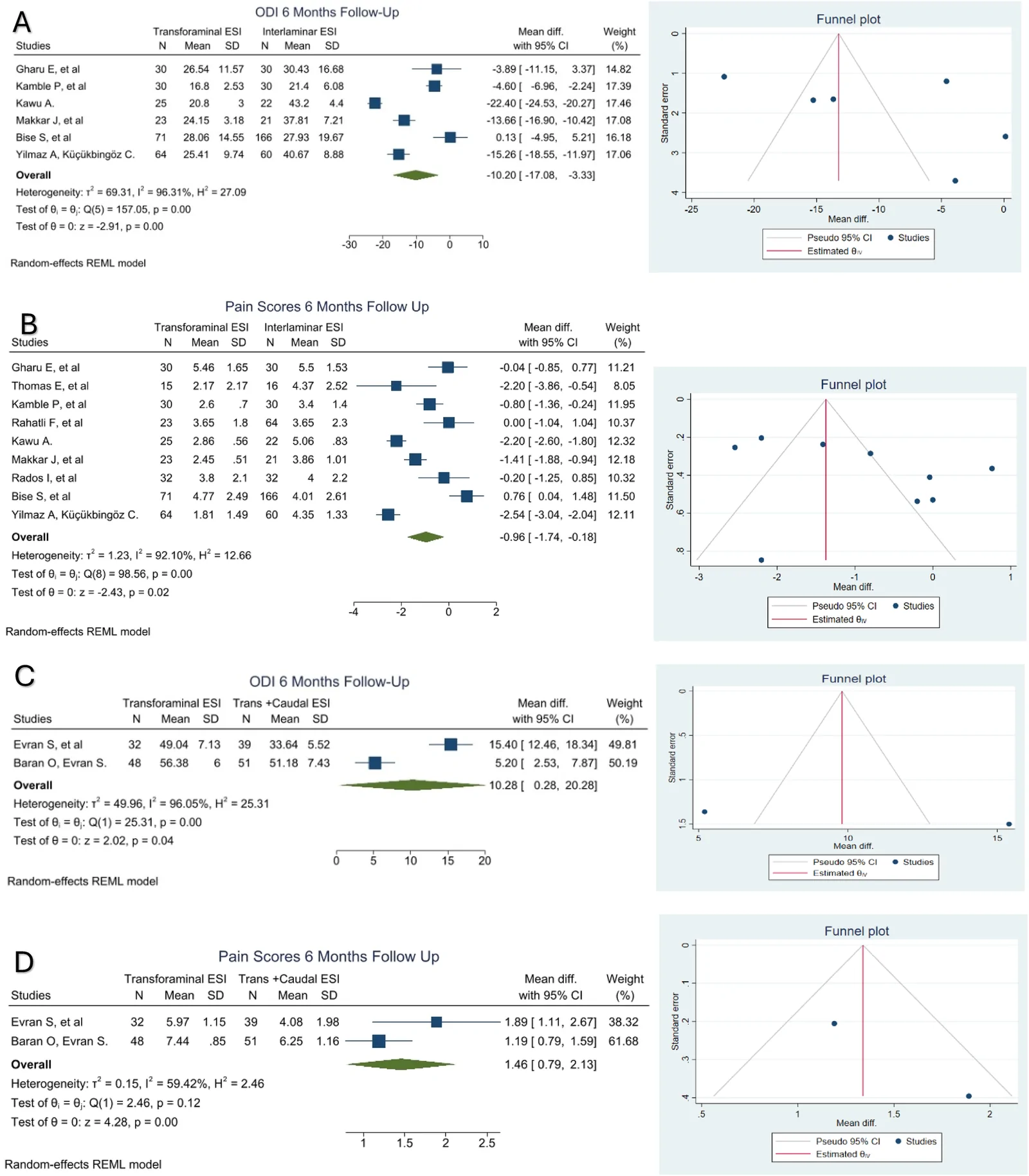
Outcomes at 6 months; **(A)** ODI scores between TFESI vs ILESI; **(B)** Pain scores between TFESI vs ILESI; **(C)** ODI scores between TFESI vs TFESI +CESI; **(D)** Pain scores between TFESI vs TFESI +CESI.

**Figure 8:**
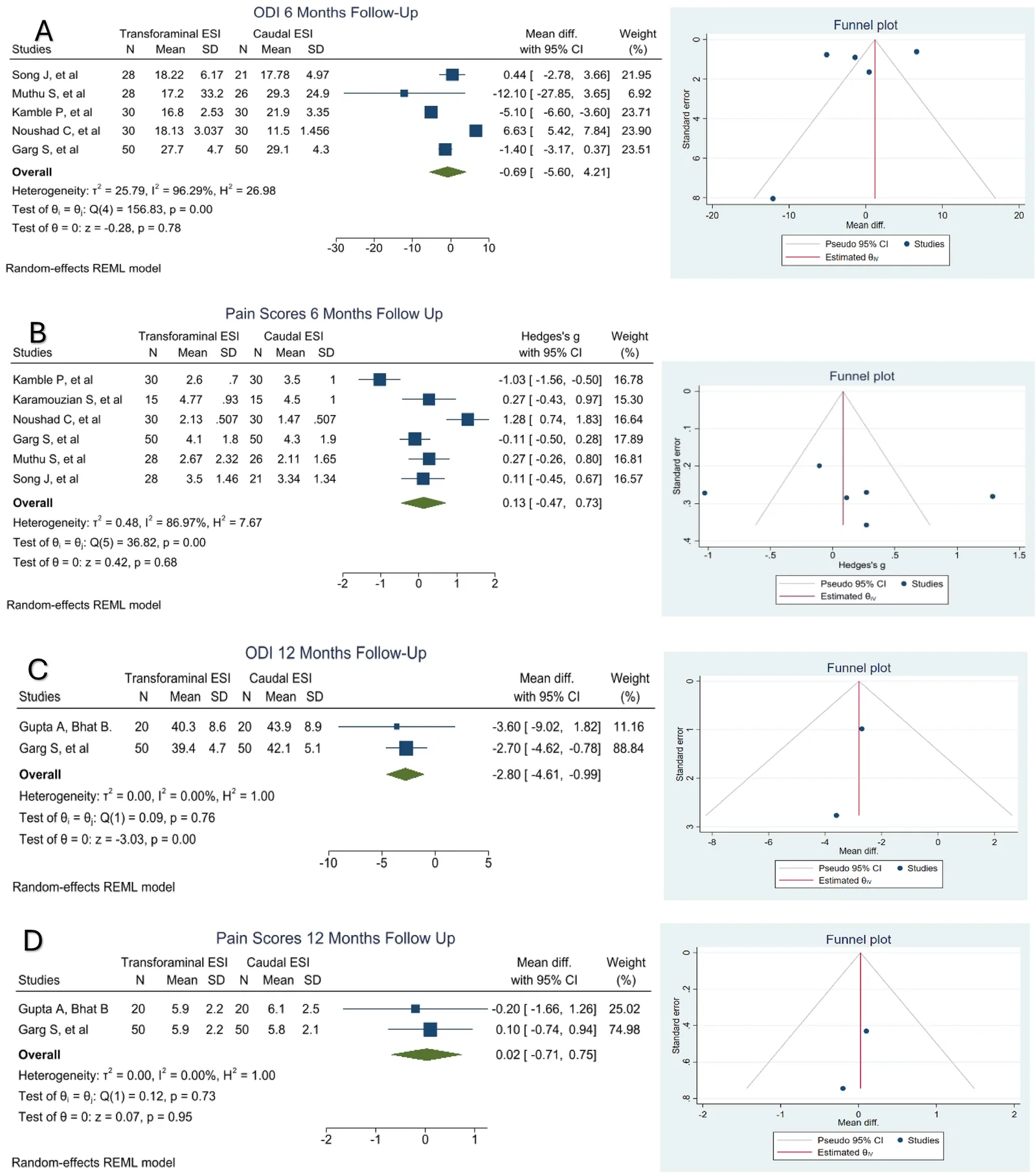
Long-term outcomes; **(A)** ODI scores between TFESI vs CESI at 6months; **(B)** Pain scores between TFESI vs CESI at 6 months; **(C)** ODI scores between TFESI vs CESI at 12 months; **(D)** Pain scores between TFESI vs CESI at 12 months

At 6-month follow-up, TFESI demonstrated a significant improvement in ODI scores (MD: −10.20; 95% CI: −17.08 to −3.33; I^2^ = 96.31%) and pain outcomes (MD: −0.96; 95% CI: −1.74 to −0.18; I^2^ = 92.10%) compared to ILESI. Similarly, the combined TFESI + CESI approach was associated with significantly greater improvement in ODI scores (MD: 10.28; 95% CI: 0.28 to 20.28; I^2^ = 96.05%) and pain scores (MD: 1.46; 95% CI: 0.79 to 2.13; I^2^ = 59.4%) compared with TFESI alone (Figure 7). However, TFESI and CESI showed similar ODI scores (MD: −0.69; 95% CI: −5.60 to 4.21; I^2^ = 96.29%) and pain scores (SMD: 0.13; 95% CI: −0.47 to 0.73; I^2^ = 86.97%) but at 12-months, TFESI showed improved ODI scores (MD: −2.80; 95% CI: −4.61 to −0.99; I^2^ = 0.00%) compared to CESI, but similar pain scores (MD: 0.02; 95% CI: −0.71 to 0.75; I^2^ = 0.00%) between the two approaches (Figure 8). Table 5 presents a summary of the main outcomes. Sensitivity analyses are presented on Supplementary Table S2, Supplementary Figure S5–S8, and SR1.

**Table 5:** Summary of the study meta-analysis results.

	Baseline		2 Weeks		1 month		3 months		6 months	
Groups	ODI scores	Pain score	ODI scores	Pain score	ODI scores	Pain score	ODI scores	Pain score	ODI scores	Pain score
TF vs IL	TF = IL	TF < IL	TF = IL	TF > IL	TF = IL	TF > IL	TF > IL	TF > IL	TF > IL	TF > IL
TF vs C	TF = C	TF = C	TF > C	TF = C	TF > C	TF > C	TF = C	TF > C	TF = C	TF = C
TF + C vs TF	TF + C = TF	TF + C = TF		TF + C > TF	TF + C > TF	TF + C > TF	TF + C > TF	TF + C > TF	TF + C > TF	TF + C > TF

TF: transforaminal, IL: interlaminar, C; caudal. The sign “=” indicates comparable outcomes between the two approaches, while the sign “>” indicates a superior outcome of one approach over another. The TF + C vs TF group did not assess outcome at the 2 weeks’ time point.

## 4 Discussion

This systematic review and meta-analysis evaluated disability and pain outcomes across ESI techniques for lumbar radiculopathy. At 2 weeks through 1 month follow-up, TFESI showed reduced pain scores compared to ILESI, but both approaches had similar ODI scores. At 3–6-month follow-up, the effect of TFESI was sustained with reduced pain scores and improved ODI scores compared to ILESI. TFESI also showed improved ODI and pain outcomes compared to CESI at early follow-up (2 weeks to 1 month). This difference was not observed in the TFESI and CESI groups between 3 and 6 months. However, the combined TFESI + CESI showed an even greater improvement in ODI and pain score compared to TFESI alone at all timepoints.

Numerous meta-analyses have assessed the efficacy of ESI for neck or back pain with varied findings (1, 4, 6, 7, 10, 13–21, 59–88), with some studies showing insufficient evidence (59, 60) or inferiority to discectomy (62), with others showing short-term pain relief and functional improvement (20, 61–65), and short-term surgery-sparing effect (17). Recent studies on ESI suggest probable pain relief and functional improvement albeit with varied levels of evidence (6, 13, 20, 21, 74, 80–83). Table 6 illustrated meta-analyses comparing the efficacy of different ESI approaches. Manchikanti et al. showed that ESI is effective with strong evidence (Level I) at one and 3 months and moderate evidence (Level II) at 6 and 12 months (80). Additionally, Zhai et al. (82) observed similar effects between epidural steroids with local anesthetics and local anesthetics alone. However, Zhao et al. observed that the effective pain relief was not statistically shown at 1 and 2 years in patients treated with lidocaine alone vs. in combination with steroids (83), suggesting a weak recommendation of steroid over local anesthetics or saline due to its remarkable superiority in pain control, especially at 1-month follow-up (21). Regarding approach, Manchikanti et al. demonstrated that ESI with local anesthetic and steroids showed Level I evidence for both transforaminal and interlaminar approaches, but Level II evidence with local anesthetic alone. In contrast, caudal epidural injections showed Level II evidence with local anesthetics with steroids or local anesthetic alone (13).

**Table 6:** Comparison of prior meta-analysis with the current study.

Studies/Year	Total number of patients	Interventional Groups	Main outcomes	Limitation of the study and Level of Evidence
Chang Chien et al. 2014 (18)	Pros: 249 (127 TFESI, 122 ILESI)	TFESI vs. ILESI	Pain outcome assessed with NRS: - The TFESI group showed greater pain relief after 2 weeks but not at 1-and 6-month follow up. - TFESI (54.1%) versus ILESI (42.7%) with <20% difference.	Studies included in analysis with: - Small number of studies included in analyses - High heterogeneity in outcomes. - No consistency or standardization of doses, injectate volumes or types of glucocorticoids. - No standardization of follow-up periods. - Variable follow-up periods mostly < 6 months. - Retrospective data not included in statistical analysis. Level of evidence: Level 1 evidence
			Disability outcome assessed with ODI and EIFEL. - ILESI group showed better functional improvement in 2 weeks - ILESI groups (56.4%) vs. TFESI groups (49.4%)	
Kwak et al. 2023 (7)	1,050 (449 TFESI, 401 ILESI, 200 CEI)	TFESI vs. ILESI vs. CESI	Pain outcomes assessed with VAS: - At short-term (1-month), TFESI > CESI, ILESI = CESI, TFESI = ILESI. However, TFEI had the highest probability of ranking first for effectiveness (77.6%), followed by CESI (51.1%) and ILESI (21.3%). - At long term (4–6months), TFESI = ILESI, ILESI = CESI, TFESI = CESI. However, TFEI had the highest probability of effectiveness (59.9%), followed by CESI (39.4%) and ILESI showed the highest probability of being least effective (66.6%).	Studies included in analysis with: - High heterogeneity in outcomes - Lack of analysis of injection volume, material, or pain onset - Authors considered results to have low evidence due to no significant difference in network meta-analysis of the long-term VAS and short- and long-term ODI changes
			Disability outcome assessed with ODI: - At short-term (1-month), TFESI = ILESI, ILESI = CESI, TFESI = CESI. However, TFEI had the highest probability of effectiveness (59.3%), followed by CESI (55.0%) and ILESI (35.7%). - At long term (4–6months), TFESI = ILESI, ILESI = CESI, TFESI = CESI. However, CESI had the highest probability of effectiveness (65.2%), followed by TFEI (56.6%) and ILESI (28.2%).	
Abdi et al. 2007 (1)	2,545 (965 TFESI, 950 ILESI, 630 CESI)	TFESI vs. ILESI vs. CESI for neck and low back pain	Study focused on level of evidence of included studies: - Pain relief short-term (<6 weeks) and long-term (> 6 weeks) - TFESI: Level II evidence for short term and Level III evidence for long-term lumbar nerve root pain, Level IV evidence for post lumbar laminectomy syndrome, Level V evidence for axial low back pain and lumbar disc extrusions. - ILESI: Level II evidence for short-term and Level IV evidence for long term relief of lumbar radiculopathy, Level V evidence for axial low back pain and lumbar spinal stenosis. - CESI: Level II evidence for short-term relief and Level III evidence for long term pain relief of lumbar radiculopathy and post-lumbar laminectomy syndrome; Level III evidence for short/long term chronic back pain.	Study limitations: - Qualitative analysis, lack of inter-group analysis Level of evidence: Variable; reported as outcomes.
Wei et al. 2016 (19)	931 (370 TFESI, 441 ILESI)	TFESI vs. ILESI	Pain outcome assessed with VAS and NRS: - VAS: TFESI > ILESI in four RCTs but TFESI = ILESI in two observation studies - NRS: TFESI > ILESI in two RCTs and one observation studies.	Studies included in analysis with: - High heterogeneity in outcomes - Lack of homogeneity in doses, injection volumes, glucocorticoids, and analgesics - Study did not include data on follow-up. Level of evidence: NR
			Disability outcome assessed with ODI: - TFESI = ILESI in four RCTs but TFESI > ILESI in one observation studies.	
Manchikanti et al. 2021 (20)	2,155 (894 TFESI, 757 ILESI, 504 CESI)	TFESI vs. ILESI vs. CESI	Pain outcomes assessed with VAS or NRS. In both TFESI and CESI groups: - Decreased pain from baseline to 6 and 12 months ILESI group: In both single and double arm studies: - Decreased pain significantly from baseline to 6 and 12 months.	Studies included in analysis with: - High heterogeneity in outcomes - Meta-analysis did not compare efficacy between groups Level of Evidence: Short and long-term: - Level I evidence for injection with local anesthetic and steroids and Level II evidence with local anesthetic alone. - For both TFESI and ILESI, Level I evidence for injection with local anesthetic and steroids and Level II evidence with local anesthetic alone for ILESI/TFESI. - Level II evidence for CESI with local anesthetic alone or for local anesthetic and steroids for short/long term relief.
			Disability outcome assessed with ODI or RMDQ: TFESI group: - Decreased ODI scores from baseline to 6 but less pronounced at 12 months ILESI group: In both single and double arm studies: - Decreased ODI scores from baseline to 6 and 12 months CESI group: - Decreased ODI scores from baseline to 6 and 12 months	
Lee et al. 2018 (21)	567	TFESI vs. CSEI	Pain outcomes assessed with VAS or NRS. - At 1 and 6 months follow up, TFESI = CESI	Studies included in analysis with: - High heterogeneity in outcomes - Small number of studies included in analyses - Authors observed that the supportive strength of this study was weak mainly because the level of evidence was low, caused by inconsistency from variation across the studies.
			Disability outcome assessed with ODI: - At 1 and 6 months follow up, TFESI = CESI Although the authors suggested TFESI had superiority over CESI, none of the mean differences reach statistical significance.	
Mahmoud et al. 2024 (14)	2,438 (1,107 men/1,119 women)	Parasagittal ILESI vs. TFESI vs. CESI	Pain outcomes assessed with VAS. For VAS 0–10 scale: Short term (< 6 months): - NMA showed that parasagittal ILESI was the most effective, followed by TFESI; however, on a head-to-head comparison, TFESI > ILESI. TFESI = CESI but became significantly superior to CESI after removing one study. Long-term (6–12months): - NMA showed that TFESI was the most effective. TFESI = ILESI. TFESI > CESI after removing one study. For VAS 0–100 scale: Short term (< 6 months): - NMA showed insignificant differences among approaches. At 1-month, TFESI was most effective but at 3 months parasagittal ILESI was most effective. TFESI = ILESI. Long-term (6–12months): - NMA showed that TFESI was the most effective, followed by parasagittal ILESI. TFESI = ILESI but became significantly higher than ILESI after removing one study.	- The superiority of TFESI over CESI was only evident after removing a study. - High heterogeneity in outcomes - Studies included employed multiple routes including parasagittal IL, middle line IL, interspinous, TF, however authors were selective in reporting their results. Level of evidence: NR
			Disability outcome assessed with ODI: Short term (< 6 months) and long-term (6–12months): - At short-term, NMA showed insignificant difference among the approaches, but at long-term TFESI was the most effective. Complications: - TFESI = parasagittal ILESI for hypotension and paresthesia risk. Risk of intravascular injection greater in TFESI	
Current meta-analysis	2,452	TFESI vs. ILESI, TFESI vs. ILESI, TFESI + CESI vs TFESI	Outcomes summarized on Table 5	Studies included in analysis with: - High heterogeneity in outcomes. - Subgroup analysis on dosing, injectate volumes, or types of glucocorticoids was not conducted. - Variable follow-up periods were mostly ≤ 6 months. Level of evidence: Variable; reported as outcomes.

NMA, network meta-analysis; the sign “=” means comparable between approaches and “>” indicate one approach is superior than the other; TFESI, transforaminal epidural steroid injections; ILESI, interlaminar epidural steroid injections; CESI, caudal epidural steroid injections; MD, mean difference; Cl, Confidence Interval; NRS, Numerical Rating Scale; VAS, Visual Analog Scale; ODI, Oswestry Disability Index; EIFEL, French translation of Roland Morris Disability Questionnaire; RCT, randomized control trial.

In our meta-analysis, TFESI demonstrated greater pain reduction than ILESI without corresponding differences in ODI scores at early follow-up (2 weeks to 1 month), a finding consistent with prior studies (Table 6) (14, 18, 19, 70). Chang-Chien et al. (18) observed that TFESI offered pain relief at 2-week follow up but no clear efficacy difference at 1 or 6 months, and Wei et al. (19) similarly reported superior short-term pain outcomes with TFESI but comparable ODI improvement. However, Mahmoud et al. observed that although the parasagittal ILESI was the most effective approach in decreasing VAS in the short term (< 6 months), the TFESI was most effective approach in decreasing both VAS and ODI in the long term (14). This supports our findings that demonstrated sustained superiority of TFESI over ILESI at 3–6 months in both pain and disability outcomes. These findings suggest that TFESI may be associated with early targeted modulation of regional inflammation and nociceptive signaling at the nerve root resulting in pain reduction; yet functional recovery may be delayed due to factors including biomechanical impairment, neuromuscular deconditioning, behavioral adaptations, or ongoing structural compression. However, as these factors gradually resolve and patients regain strength and mobility, functional improvements become more clinically apparent over time. For ILESI, heterogeneity in reported efficacy across studies may reflect variability in injection technique (e.g., midline vs. parasagittal approach), injectate spread within the epidural space, etc. Despite this variability, Gill et al. (70) demonstrated the greatest ODI reduction at 6 months following ILESI, suggesting that ILESI can provide meaningful functional improvement in appropriately selected patients.

For TFESI versus CESI, our analyses demonstrated an early advantage for TFESI, with improved disability at 2 weeks and both pain relief and disability reduction by 1 month, consistent with prior reports (21, 70). Although CESI has been shown to provide both short and long-term pain relief (71) and was associated with the highest ODI improvement at 1 month (70) in one study, other reports suggest that its overall effect may be inferior to TFESI (21, 70). At 3-month follow-up, the TFESI group demonstrated significant pain relief but comparable disability outcomes as CESI. Also, Lee et al. reported superiority of TFESI over CESI in lumbosacral disc herniation, suggesting this difference to TFESI's ability to deliver medication directly into the target area, whereas CESI may require a larger amount of medication to be effective (21). Mechanistically, TFESI facilitate targeted delivery to the ventral epidural space, nerve root sleeve, and disc–nerve interface, optimizes pharmacologic concentration at the site of radicular pathology, while CESI relies on diffuse epidural spread, longer diffusion distances, and potential dilutional effects, which may attenuate its focal efficacy. Moreover, Parr et al. demonstrated that the effectiveness of CESI may vary by etiology. While CESI may provide meaningful short- and long-term pain relief in cases of disc herniation or radiculitis, its efficacy in chronic axial or discogenic pain, spinal stenosis, and post-surgical syndrome appears to be only fair (72). Accordingly, the caudal approach was favored in older studies, but more recent literature has increasingly favored the transforaminal route (87).

Despite these findings, we observed that by 6 months follow-up, pain and ODI outcomes between TFESI and CESI converged. At the 12-month follow-up, our analysis suggested that patients undergoing TFESI may maintain greater improvements in disability compared with those treated with CESI; however, both groups demonstrated comparable levels of pain relief at this timepoint. This pattern may reflect delayed therapeutic effects of CESI through cumulative epidural diffusion and multi-segmental anti-inflammatory activity, while TFESI effects may plateau after early benefit. Besides plateauing of TFESI effect overtime, the efficacy of this approach is not uniform across all studies. For instance, one meta-analysis showed that TFESI provided pain relief at 3 months but had no impact on physical disability or incidence of surgery (76). Other meta-analyses have demonstrated significant pain reduction at 3–6 months without corresponding improvements in back-specific disability (77, 78). Furthermore, Pairuchvej et al. reported that preganglionic TFESI was associated with a 2.38-fold higher likelihood of effectiveness compared with ganglionic TFESI, suggesting that technical nuances within the transforaminal approach itself may influence outcomes (79).

Despite this variability, our findings indicate that combined TFESI + CESI produced greater and more sustained improvements in both pain and ODI scores from 1 through 6 months of follow-up compared with TFESI alone. The observed benefits of combined TFESI + CESI introduce a novel therapeutic paradigm not well characterized in previous comparative studies. This pattern suggests complementary mechanisms, focal nerve-root modulation via TFESI combined with diffuse multilevel epidural anti-inflammatory effects via CESI. The additive distribution profile may better address both localized radicular pathology and broader epidural inflammatory processes.

Although subgroup analyses were not performed in this study, previous studies have shown variability in therapeutic outcomes regardless of ESI approach (84, 85). In one study, the use of particulate steroids was associated with slightly better VAS scores only (84). In another report, methylprednisolone showed better effect compared with dexamethasone, while dexamethasone appeared more effective than betamethasone. Despite these differences, the authors recommended using non-particulate steroids for lumbar radicular pain due to their safety profile (85). From a safety perspective, recent evidence suggests that ultrasound guided ESIs are not only comparable to conventional fluoroscopy-guided ESIs in terms of pain control and functional improvement but also have a lower risk of inadvertent vascular puncture (88). Considering approach, Lee et al. observed that ultrasound-guided TFESI featured less intravascular leakage of contrast than ILESI albeit the evidence level was low due to inconsistency and imprecision (68). Additionally, another meta-analysis demonstrated that compared to real-time fluoroscopy, digital subtraction angiography had a 32% improvement for detection of intravascular penetration with ESI and advocated for the increased use of DSA over real-time fluoroscopy for TFESIs (86). Notably, however, higher detection rates of vascular penetration with real-time fluoroscopy did not correlate with increased cumulative complication rates, which remained low (approximately 1%). Collectively, these findings underscore that both pharmacologic factors (e.g., steroid type and particulate properties) and technical considerations may influence both efficacy and safety outcomes following the different ESI approaches.

## 5 Limitations

This meta-analysis has several limitations. First, we did not perform subgroup analyses or meta-regression to further explore heterogeneity among the included studies with respect to study characteristics, steroid type, dosage, injection protocols, imaging guidance, underling pathology and outcome measures because these variables were inconsistently reported across studies. For example, although some studies reported the number of repeated injections administered, many did not. Similarly, fluoroscopic guidance was used in most studies, providing insufficient variability to evaluate its influence on treatment outcomes. Although the steroid type was reported by individual studies, most used similar dosing protocols between treatment groups, whereas others used different formulations or doses, limiting our ability to perform meaningful subgroup analyses or meta-regression. Second, follow-up durations varied across studies, and adverse event reporting was inconsistent. Additionally, high-quality RCTs directly evaluating combined vs. single-technique strategies remain lacking; thus, the results should be interpreted with caution. Third, there was inconsistency in the time points at which outcomes were reported across studies. In some cases, we approximated follow-up intervals to harmonize data synthesis. To mitigate misrepresentation, results were presented within defined time windows to capture temporal trends while acknowledging this limitation. Fourth, although different pain assessment tools were used across studies, these instruments measure similar constructs and are considered clinically interchangeable. Nevertheless, residual heterogeneity regarding measurement methods cannot be completely excluded. Despite these limitations, the available evidence synthesized in this meta-analysis demonstrated an additive benefit of combined TFESI + CESI compared with TFESI alone. These results suggest a potential role for multimodal injection strategies in patients with refractory radiculopathy, significant functional limitation, or complex degenerative pathology.

## 6 Conclusion

To our knowledge, this is the first meta-analysis of ESIs to evaluate the combined effect of TFESI + CESI compared with other approaches in patients with lumbar radiculopathy. Our results indicate that although TFESI demonstrates a clinically meaningful advantage over ILESI and CESI, this effect is not entirely consistent across all follow-up time points. TFESI appears to provide earlier pain relief and sustained functional improvement; however, the combined TFESI + CESI approach offers additive clinical effects that may exceed those of single-technique interventions. These findings support TFESI as a preferred first-line interventional strategy while highlighting the emerging role of combined techniques in carefully selected patient populations.

## Data Availability

The original contributions presented in the study are included in the article/Supplementary Material, further inquiries can be directed to the corresponding author.
