# Supplementary material for: Effectiveness of epidural steroid injections in the treatment of lumbar radiculopathy: a systematic review and meta-analysis
Source: Front Pain Res (Lausanne). 2026 Aug 21;7:1904875. doi: 10.3389/fpain.2026.1904875 (PMC13541943; doi:10.3389/fpain.2026.1904875)
Supplement: Supplementary file 1 [file Table1.docx]

**Supplementary results**

Supplementary results 1:

**Sensitivity Analysis**

Overall, the pooled estimates were moderately stable, especially at later follow-up intervals. For TFESI vs ILESI, ODI outcomes were very robust at baseline and 6 months and moderately robust at 3 months, while pain score estimates were moderately to very robust at 2 weeks - 1 month but only robust at baseline and 6 months but showed some instability 3 months (Figures S5 and S6). For TFESI vs CESI, ODI estimates were very robust at baseline and 1 month and moderately robust at 2 weeks but unstable at 3-6 months, whereas pain outcomes were generally very robust at baseline and 6 months but moderately stable at other timepoints (Figures S7 and S8; Table S3). Sensitivity analysis was not feasible for TFESI vs TFESI +CESI due to the limited number of studies.

**Publication Bias**

Based on the Egger’s test, publication bias (PB) for various outcomes was low. In the TFESI and ILESI comparison groups, for ODI scores, there was no PB at baseline (t= -0.15, p=0.846), 2 weeks follow-up (t= 0.09, p=0.965), 1 month follow-up (t= 2.35, p=0.050), 3 months follow-up (t= 0.37, p=0.931), 6 months follow-up (t= 5.47, p=0.123). For pain scores, no PB at baseline (t= -0.06, p =0.953), 2 weeks follow-up (t= -8.54, p=0.119), 1 month follow-up (t= 0.31, p=0.788), 3 months follow-up (t= -0.07, p=0.981), 6 months follow-up (t= 1.16, p=0.624). In the TFESI and CESI comparison group, for ODI scores, there was no PB at baseline (t= -1.54, p=0.192), 2 weeks follow-up (t= 2.86, p=0.248), 1 month follow-up (t= 0.26, p=0.847), 3 months follow-up (t= -0.18, p=0.905), 6 months follow-up (t= -1.73, p=0.289). For pain scores, no PB at baseline (t= -0.19, p=0.925), 2 weeks follow-up (t= -9.71, p=0.453), 1 month follow-up (t= -3.29, p=0.619), 3 months follow-up (t= 2.05, p= 0.773), 6 months follow-up (t= 3.13, p=0.689). Due to limited studies, PB was not assessable for the TFESI vs TFESI+CESI group.

**Table S1 – Search strategy**

| **Databases searched:** | **Search string used:** | **Number of articles** |
| --- | --- | --- |
| PubMed | #1 (("lumbar radiculopathy"[All Fields] OR "sciatica"[All Fields]) AND "epidural steroid injection"[All Fields]) OR "transforaminal epidural steroid injection"[All Fields] OR "interlaminar epidural steroid injection"[All Fields] OR "caudal epidural steroid injection"[All Fields] OR "TFESI"[All Fields] OR "CESI"[All Fields] OR "ILESI"[All Fields] | 1,614 articles |
| Scopus | #1 ALL "lumbar radiculopathy" OR "sciatica" AND "epidural steroid injection" OR "transforaminal epidural steroid injection" OR "interlaminar epidural steroid injection" OR "caudal epidural steroid injection" OR "TFESI" OR "CESI" OR "ILESI" AND ( LIMIT-TO ( DOCTYPE , "ar" ) ) | 2,230 articles |
| Cochrane | #1 "lumbar radiculopathy" OR "sciatica" AND "epidural steroid injection" OR "transforaminal epidural steroid injection" OR "interlaminar epidural steroid injection" OR "caudal epidural steroid injection" OR "TFESI" OR "CESI" OR "ILESI":ti,ab,kw | 619 articles |
| **Total** | | 4,463 articles |

**Search updated:** 12/16/2025

**Total:** 4,463; **Duplicates:** 940; 3,523 to screen.

**Table S2 - Sensitivity analysis for study robustness**

| **TFESI vs ILESI** | | | | |
| --- | --- | --- | --- | --- |
|  | ODI | Interpretation | Pain scores | Interpretation |
|  | MD (95% CI), p value |  | MD (95% CI), p value |  |
| Timepoints |  |  |  |  |
| Baseline | 0.02 (-1.08 to 1.11), 0.98 | Very robust | –0.16 (–0.27 to –0.04), 0.01 | Robust |
| 2weeks | −0.33 (−4.18 to 3.52), 0.87 | Unstable | –1.73 (–1.14 to –0.32), 0.001 | Very robust |
| 1 month | -3.35 (−7.42 to 0.73), 0.11 | Unstable | –0.64 (–1.13 to –0.15), 0.01 | Moderately robust |
| 3 months | −4.92 (−9.57 to −0.28), 0.04 | Moderately robust | –0.58 (–1.11 to –0.04), 0.04 | Unstable |
| 6 months | −10.20 (−17.08 to −3.33), 0.004 | Very robust | –0.96 (–1.74 to –0.19), 0.02 | Robust |
| **TFESI vs CESI** | | | |  |
| Baseline | −0.60 (−2.50 to 1.31), 0.54 | Very robust | 0.03 (−0.11 to 0.17), 0.71 | Very robust |
| 2weeks | −4.34 (−7.64 to −1.05), 0.01 | Moderately robust | −0.77 (−1.78 to 0.24), 0.14 | Moderately robust |
| 1 month | −3.82 (−6.35 to −1.29), 0.003 | Very robust | −0.45 (−0.87 to −0.03), 0.04 | Moderately robust |
| 3 months | −0.20 (−3.80 to 3.40), 0.92 | Unstable | −0.50 (−0.96 to -0.03), 0.04 | Moderately robust |
| 6 months | −0.70 (−5.60 to 4.21), 0.78 | Unstable | 0.13 (−0.47 to 0.73), 0.68 | Very robust |
| 12 months* |  |  |  |  |
| **TFESI vs TFESI + CESI*** | | | | |

*Only 2 studies were identified therefore leave-one-out test was not performed at this timepoint. MD; mean difference, CI; confidence interval
