# Supplementary material for: Effectiveness of epidural steroid injections in the treatment of lumbar radiculopathy: a systematic review and meta-analysis
Source: Front Pain Res (Lausanne). 2026 Aug 21;7:1904875. doi: 10.3389/fpain.2026.1904875 (PMC13541943; doi:10.3389/fpain.2026.1904875)
Supplement: Supplementary file 2 [file Table2.docx]

**Supplementary Figures**

**Figure S1. Postoperative 2 Weeks ODI and Pain Scores; TFESI vs ILESI**

**Figure S2. Postoperative 2 Weeks ODI and Pain Scores; TFESI vs CESI**

**Figure S3. Postoperative 1 month ODI and Pain Scores; TFESI vs TFESI +CESI**

**Figure S4. Postoperative 3 months ODI and Pain Scores; TFESI vs CESI**

Figure S5; Sensitivity analysis with leave one out test showing ODI scores at; (A) baseline, (B) 2 weeks follow-up, (C) 1 month follow-up, (D) 3 months follow-up, and (E) 6 months follow-up in the TFESI versus ILESI comparison groups.

Figure S6; Sensitivity analysis with leave one out test showing pain outcome scores at; (A) baseline, (B) 2 weeks follow-up, (C) 1 month follow-up, (D) 3 months follow-up, and (E) 6 months follow-up in the TFESI versus ILESI comparison groups.

Figure S7; Sensitivity analysis with leave one out test showing ODI scores at; (A) baseline, (B) 2 weeks follow-up, (C) 1 month follow-up, (D) 3 months follow-up, and (E) 6 months follow-up in the TFESI versus CESI comparison groups.

Figure S8; Sensitivity analysis with leave one out test showing pain outcome scores at; (A) baseline, (B) 2 weeks follow-up, (C) 1 month follow-up, (D) 3 months follow-up, and (E) 6 months follow-up in the TFESI versus CESI comparison groups.
