# Supplementary material for: Effectiveness of epidural steroid injections in the treatment of lumbar radiculopathy: a systematic review and meta-analysis
Source: Front Pain Res (Lausanne). 2026 Aug 21;7:1904875. doi: 10.3389/fpain.2026.1904875 (PMC13541943; doi:10.3389/fpain.2026.1904875)
Supplement: Supplementary file 3 [file Table3.docx]

**Abbreviations**

ESI: Epidural steroid injection

TFESI: Transforaminal epidural steroid injection

ILESI: Interlaminar epidural steroid injection

CESI: Caudal epidural steroid injection

PRISMA: Preferred Reporting Items for Systematic Reviews and Meta-Analyses

ODI: Oswestry disability index

VAS: Visual analogue scale

NRS: Numeric rating scale

NPRS: Numeric pain rating scale

VNS: Visual numerical scale scores

RoB: Risk of Bias

RCT: Randomized clinical trials

MD: Mean differences

CI: Confidence intervals

MJOA: modified Japanese Orthopedic Association scale

GPE: Global Perceived Effect questionnaire

RMDQ: Roland Morris Disability Questionnaire

DPQ: Dallas pain questionnaire,

RMLBPQ: Roland-Morris low back pain questionnaire

DN4: Neuropathic Pain Component

CORI: Clinical Outcome Rating Index

MRM: Modified Roland-Morris

NASS: North American Spine Society pain satisfaction index

EQ‑5D: EuroQol five dimensions quality of life scores

OLBPS: Oswestry low back pain scale

BDS: Beck depression scores

ICIDH-1: WHO-modified International Classification of Impairments, Disabilities and Handicaps
